# A focus on anionic boron anthracenes and triptycenes as entry point toward B-doped polyaromatic materials and Lewis acids

**DOI:** 10.1039/d3sc90074f

**Published:** 2023-05-09

**Authors:** Guillaume Berionni

**Affiliations:** a Université de Namur, Department of Chemistry, Namur Institute of Structured Matter Rue de Bruxelles 61 5000 Namur Belgium guillaume.berionni@unamur.be

## Abstract

This article highlights the recent work of M. Wagner and collaborators on the synthesis, bridgehead functionalization, and photoisomerization of boron-doped triptycene derivatives (https://doi.org/10.1039/D3SC00555K).

## Graphical abstract

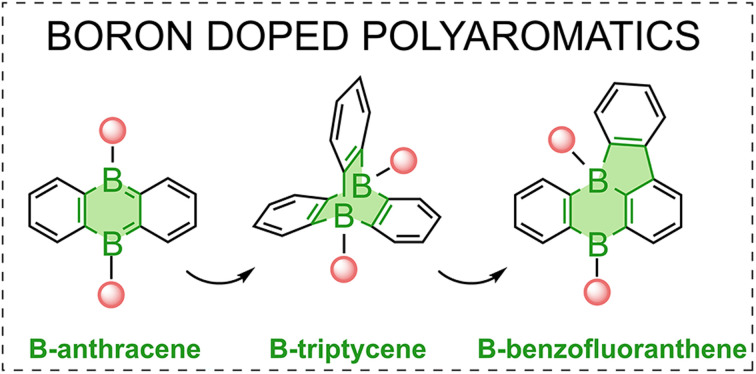

Main-group element doped polycyclic aromatics and heterocycles are of considerable practical value, as they expand the panel of electronic structures, photophysical properties and chemical reactivity of conventional polyaromatic hydrocarbons.^1^ Boron-doped aromatic materials based on the anthracene, fluorene and triptycene cores are increasingly used for applications in organic electronics, functional materials, polymers and anion sensors.^2^ Owing to the cooperative reactivity between their two boron atoms (Scheme 1), dual boron-doped dianionic acenes exhibit transition metal-like versatile reactivities toward small molecules (*e.g.* O_2_, CO, CO_2_, H_2_, alkenes, carbonyls, silanes).^3^

**Scheme 1:**
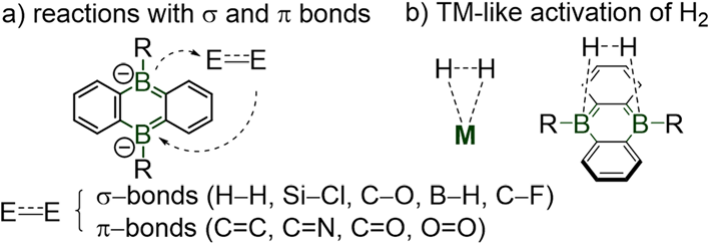
Prominent examples of reactions of diboraanthracene with small molecules through boron cooperation.^3^

Recent efforts by Wagner and collaborators led to the first doping of the two bridgehead positions of the unique paddle-wheel shaped triptycene *D*_3h_-symmetric scaffold by two boron atoms.^4^ Analogously to the seminal Wittig synthesis of triptycene 2 by cycloaddition between anthracene 1 and *in situ* generated benzyne (Scheme 2a),^5^ Wagner *et al.* have obtained a series of diboratatriptycene dianions 4-R by reacting benzyne with the respective anionic diboraanthracenes 3-R (Scheme 2b).

**Scheme 2:**
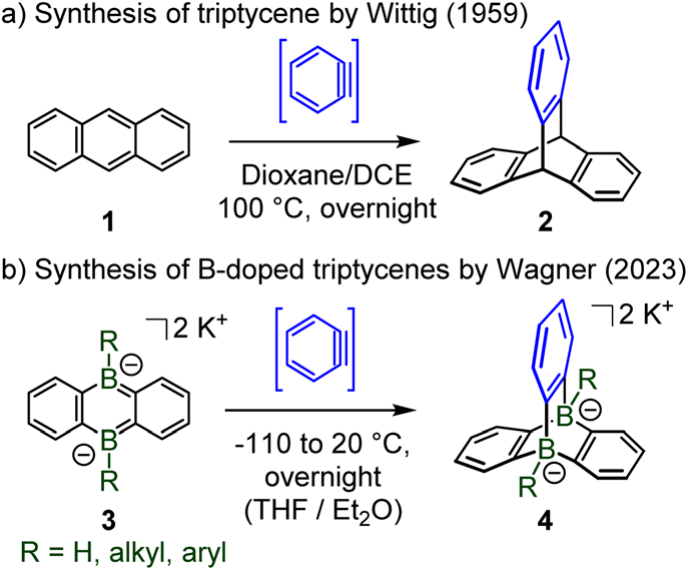
(a) Synthesis of triptycene by [4 + 2] cycloaddition; (b) synthesis of dihydride, dialkyl and diaryl 9,10-diboratatriptycene dipotassium salts.

Both bridgehead borohydrides in 4-H undergo B–H/B–Cl exchange by reaction with dichloromethane, providing the bischloroborate triptycene 4-Cl (Scheme 3a). Reaction of 4-H with tris(pentafluorophenyl)borane B(C_6_F_5_)_3_, a widely used boron Lewis acid in main group chemistry, in combination with dimethylsulfide as Lewis base, resulted in the formation of the neutral ditopic Lewis adduct 4-SMe_2_ (Scheme 3a). Quantum-chemical calculations highlighted that the pyramidalized trivalent boron Lewis acids which are formed transiently during these transformations are particularly strong Lewis acids, as judged by their computed F^−^ ion affinities (gas phase values, Scheme 3b) reaching that of B(C_6_F_5_)_3_ (FIA = 466 kJ mol^−1^)^6^ and that of the previously reported 9-boratriptycene (FIA = 476 kJ mol^−1^).^6^

**Scheme 3:**
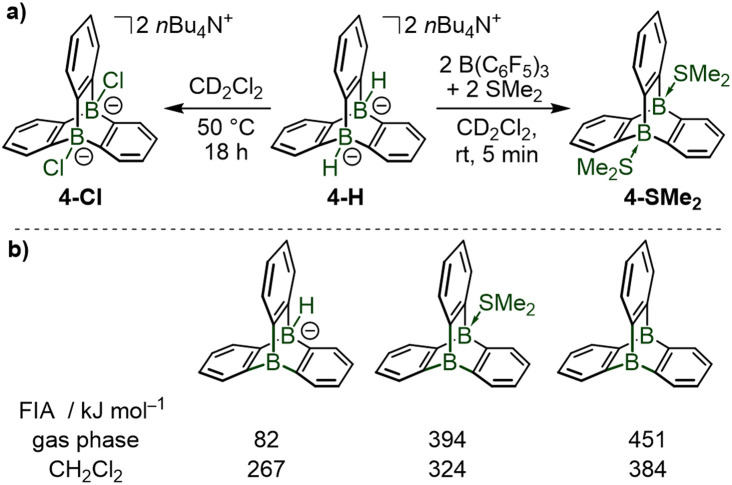
Reactivity of the 9,10-diboratatriptycene dihydride 4-H: activation of the B–H bonds and coordination of the bridgehead boron atoms with anions or Lewis bases *via* the generation of transient pyramidal boron Lewis acids. Fluoride ion affinities computed at the PBE0-D3(BJ)/def2-TZVPPD theory level using isodesmic reactions with COF_2_ anchor point, values from ref. 4.

A key advance in boron chemistry is the photorearrangement of the diboratotriptycene 4-H into a diborabenzo[*a*]fluoranthene 5 which combines the two prominent structural motifs of 9,10-dihydro-9,10-diboraanthracene and 9*H*-9-borafluorene (Scheme 4a). Such photorearrangements are following similar cascade steps as in the corresponding all-carbon triptycene derivative 2, which was also re-investigated experimentally and computationally by Wagner in this study.^4^ The single other method to access a diborabenzofluoranthene compound 7, which was recently described by Ji, Lin, Braunschweig and Marder,^7^ is involving a relatively long thermally driven rearrangement of the *ortho*-phenyl bis-9-borafluorene 6 at an elevated temperature (Scheme 4b).^7^

**Scheme 4:**
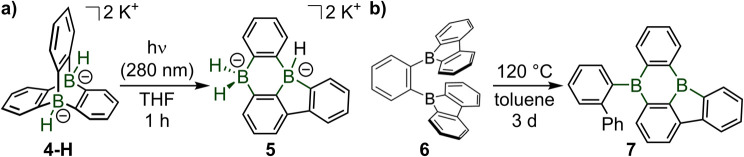
Two known examples of diborabenzo[*a*]fluoranthene, 5 and 7.

Major impact in main-group chemistry and coordination chemistry is expected, since combining ditopic Lewis acids with ditopic Lewis bases will provide access to new inorganic polymers with alternating triptycene and Lewis base spacer. As diboratriptycene dianions are featuring a unique orientation of their aryl group, they act as chelating ligands toward M^+^ ions *via* the π-electron clouds of the two 1,2-phenylene rings,^4^ showcasing further use of these cage-shaped anionic di-boronate-triptycenes for the coordination of metallic cations.

The most direct application is the production of pyramidal boron Lewis acids and superacids with promising structures and reactivities, which have recently been proven to perform the C–H borylation of unreactive aromatics,^8^ and that can be embedded in other pyramidal frameworks connected to the ferrocene scaffold.^9^

## Author contributions

G. Berionni wrote the article.

## Conflicts of interest

There are no conflicts of interests to declare.

## Supplementary Material
